# Editorial: Robotic *In-Situ* Servicing, Assembly and Manufacturing

**DOI:** 10.3389/frobt.2022.887506

**Published:** 2022-03-31

**Authors:** Craig R. Carignan, Renaud Detry, Mini Chakravarthini Saaj, Giacomo Marani, Joshua D. Vander Hook

**Affiliations:** ^1^ Department of Aerospace Engineering, University of Maryland, College Park, MD, United States; ^2^ Institute for Artificial Intelligence, KU Leuven, Leuven, Belgium; ^3^ School of Engineering, University of Lincoln, Lincoln, United Kingdom; ^4^ West Virginia Robotic Technology Center, West Virginia University, Morgantown, WV, United States; ^5^ NASA Jet Propulsion Laboratory (JPL), La Cañada Flintridge, CA, United States

**Keywords:** space robotics, in-space assembly, robotic servicing, cooperative control, robotic inspection, in-space manufacturing, in-situ sensing, navigation

Conceptual designs for future space science and commercialization efforts are featuring ever larger structural assemblies. These projects include large space telescopes, radio frequency antennae, solar power stations, and persistent platforms for satellite servicing and commercial space markets. NASA has even explicitly called for on-orbit autonomous assembly and aggregation of large space structures, modular design, and interfaces to meet the agency’s future science and exploration needs. Similar initiatives are also now supported by the European Space Agency and the UK Space Agency. Future applications include *in-situ* resource extraction and utilization, habitat construction, and monitoring and servicing of infrastructure.

**FIGURE 1:**
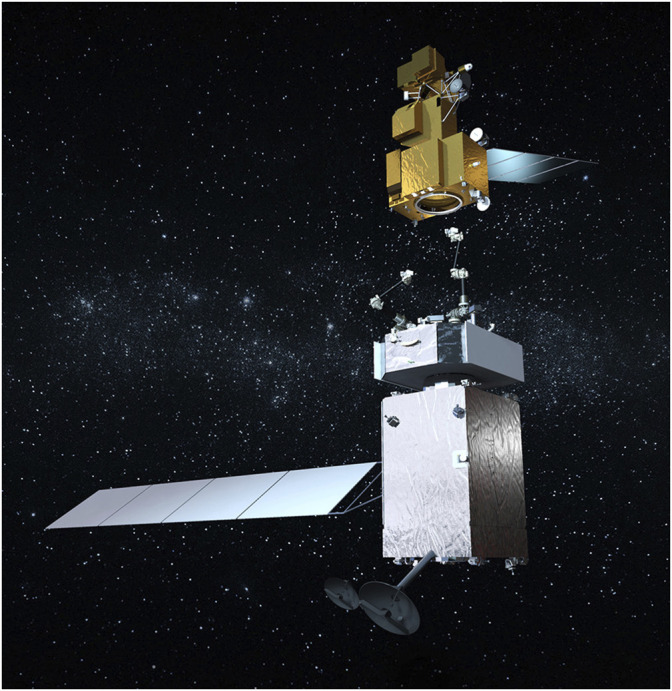
NASA’s OSAM-1 Mission will capture, refuel, and relocate the LandSat7 satellite. Credit: NASA.

Several aerospace companies and smaller start-up companies are proposing systems to fabricate and integrate large structures on orbit using robots with the goal of beginning on-orbit manufacturing and assembly of large structures within the next few years. NASA and Maxar, Inc. are teaming up on the OSAM-1 Mission to equip a robotic spacecraft with a pair of dexterous robot arms to capture, refuel, and relocate a government-owned satellite to extend its life (see Figure 1). Tether Unlimited’s “SpiderFab” consists of a robotic system that builds up large, sparse structures by extruding high performance structural elements and assembling them into a larger structure. Redwire Space’s “Archinaut One” robot (now OSAM-2) will build, assemble, and deploy its own solar array. These robotic technologies will enable the construction and deployment of systems that require large apertures or baselines using much smaller and less expensive launch vehicles thus reducing life cycle cost.

This research topic is dedicated to articles focused on robotic manufacturing, assembly, and servicing utilizing *in-situ* resources, especially for space robotic applications. Several authors featured here also presented at the “Robotic *In-Situ* Servicing, Assembly and Maintenance” workshop held at the 2020 IEEE/RSJ International Conference on Intelligent Robots and Systems (IROS 2020 http://iros2020.org) in Las Vegas, Nevada United States on 25 October 2020 (https://wvrtc.com/iros2020). The purpose was to bring together researchers from a variety of disciplines to identify common themes, formulate problems, and share promising technologies for autonomous large-scale construction, servicing, and assembly robots. The articles under this special topic provide a snapshot of several key technologies under development to support on-orbit robotic servicing, assembly, and manufacturing.

One of the key technical challenges in working with space robots is their high flexibility afforded by the much lower mass of their earth-based counterparts which need to endure gravitational loading. Holmes of MacDonald Dettwiler Associates tackles potential control instability posed by the low frequency natural dynamics. Classical control strategies are brought to bear on both collocated (joint rate) and non-collocated (force/torque and vision-based) feedback approaches. The analysis indicates additional constraints on the design control bandwidth to maintain stability lending additional validation to popular heuristic methods.

Carabis and Wen at Rensselaer Polytechnic Institute tackle the challenges of designing trajectories for space manipulators with joint flexibility and limited joint torque, particularly when manipulating large objects such as satellites during servicing activities. Their model-based trajectory generation methodology incorporates constraints on joint speed, motor torque, and base actuation for flexible-joint space manipulators while minimizing total trajectory time. Full spatial simulation results are compared with experimental results performed on an air-bearing table to demonstrate the efficacy of their approach.

Planning and directing in-space tasks range from direct human intervention through teleoperation to fully autonomous agents. Kazanzides et al. at the Johns Hopkins University Laboratory for Computational Sensing and Robotics explore control and visualization interfaces for operators performing tasks using direct and semi-autonomous teleoperation. The improved visualization and situational awareness afforded by their model-based architecture enable operators to precisely specify intended motion during satellite servicing tasks subject to round-trip communication latencies of several seconds.

Moser et al. at Virginia Tech’s Field and Space Experimental Robotics Lab imbue space robots with autonomous capability for maintaining and repairing in-space and planetary surface assets without the need for human intervention. A stochastic problem formulation paired with a mixed integer programming assembly schedule generator is developed to produce an optimal assembly schedule. Experiments demonstrate the capability of the formulation and optimization methodologies and highlight the system’s ability to resequence and reallocate after assembly is already underway.

Stationary versus mobile robotic platforms is a key tradeoff especially in on-orbit assembly. McBryan at the U.S. Naval Research Laboratory compares stationary and mobile robots equipped with a pair of dexterous manipulators for performing in-space assembly of a truss structure. The analysis details the interplay between the mass of the structure and the requirements of the robotic system. A task sequencing and allocation optimization method was also developed in conjunction with the study to generate optimal task sequences and robot-task allocations to optimize the assembly.

Seddaoui et al. at the Surrey Space Centre and the Lincoln Centre for Autonomous Systems state that spacecraft mounted with one or more robotic manipulators is “inevitable” for a range of future in-orbit services. However, controlling a space robot in free-flying and free-floating modes is very challenging, and this tutorial article aims to address the knowledge gap in modeling complex space robots operating in the controlled-floating mode and under perturbed conditions. The nonlinear model developed accurately captures the multibody coupled dynamics of a space robot, and simulation results demonstrate the accuracy of the model for closed-loop control.

Reliability of robot manipulators deployed in hazardous environments when a failure occurs is a key concern especially when humans are nearby. Porges et al. at the German Aerospace Center examine the potential risk and mitigation strategies in the event of a joint failure of a kinematically redundant manipulator. While their methodology is based on off-line pre-computed workspace models (and is thus not real-time), it is general enough to cope with robots with any type and number of joints and might include arbitrarily shaped obstacles in the process.

We hope that you will enjoy this special issue and will consider contributing to a future research topic or workshop on robotic on-orbit servicing, assembly, and manufacturing!.

